# Clinical ascertainment of health outcomes in Asian survivors of childhood cancer: a systematic review

**DOI:** 10.1007/s11764-019-00759-9

**Published:** 2019-05-04

**Authors:** Long Hin Jonathan Poon, Chun-Pong Yu, Liwen Peng, Celeste Lom-Ying Ewig, Hui Zhang, Chi-Kong Li, Yin Ting Cheung

**Affiliations:** 1 School of Pharmacy, Faculty of Medicine, The Chinese University of Hong Kong, 8th Floor, Lo Kwee-Seong Integrated Biomedical Sciences Building, Shatin, N.T Hong Kong; 2 Li Ping Medical Library, The Chinese University of Hong Kong, Hong Kong, Hong Kong; 3 Department of Pediatric Hematology/Oncology, Guangzhou Women and Children’s Medical Center, Guangzhou, China; 4 Department of Paediatrics, Faculty of Medicine, The Chinese University of Hong Kong, Hong Kong, Hong Kong; 5 Paediatric Haematology & Oncology, Hong Kong Children’s Hospital, Hong Kong, Hong Kong

**Keywords:** Childhood cancer, Late effects, Survivorship, Risk-based, Asian, Organ toxicity

## Abstract

**Purpose:**

Survivorship in children with cancer comes at a cost of developing chronic treatment-related complications. Yet, it is still an under-researched area in Asia, which shares the largest proportion of the global childhood cancer burden given its vast population. This systematic review summarizes existing literature on clinically ascertained health outcomes in Asian survivors of childhood cancer.

**Methods:**

A search was conducted on Ovid Medline and EMBASE for studies that focused on survivors of childhood cancer from countries in East and Southeast Asia; adopted post-treatment clinical ascertainment of organ-specific toxicities or/and secondary malignancy. Studies were excluded if health outcomes were assessed during the acute treatment.

**Results:**

Fifty-nine studies, enrolling a total of 13,442 subjects, were conducted on survivors of leukemia (34%), CNS tumor (14%), and cohorts of survivors with heterogeneous cancer diagnoses (52%). The studies used different medical evaluation methods to assess cardiovascular (15%), metabolic and infertility (32%), and neurological/neurocognitive (20%) outcomes in survivors. The collective findings suggest potential differences in the prevalence of certain late effects (e.g., secondary malignancy and obesity) among Asian and non-Asian populations, which may reflect differences in treatment regimens, practice, genetic variations, or/and socioeconomic disparity.

**Conclusions:**

We recommend developing collaborative initiatives to build a regional repository of systematically assessed health outcomes and biospecimens to investigate treatment, social-environmental and genetic predictors, and interventions for late effects in this population.

**Implications for Cancer Survivors:**

The existing types of chronic health problems identified in this review suggest the need for active screening, better access to survivorship care, and promotion of protective health behavior in Asia.

**Electronic supplementary material:**

The online version of this article (10.1007/s11764-019-00759-9) contains supplementary material, which is available to authorized users.

## Introduction

Childhood cancer is an emerging priority in the current global child health agenda. Given its vast population, Asia shares the largest proportion of the global childhood cancer burden. A recent report by the International Agency for Research on Cancer (IARC) indicated that the childhood cancer burden in Asia now accounts for approximately 50% of all cases worldwide, and this proportion is expected to increase in the coming decades [1, 2].

The development of modern treatment strategies has led to tremendous improvements in the 5-year survival and cure rates of childhood cancer during the past 40 years, particularly in high-income Asian countries such as Japan, Singapore, South Korea, and Hong Kong, where survival rates are mostly above 80%, comparable to that of the Western population [3–6]. However, survival rates in low-income and middle-income countries of Asia (e.g., mainland China, the Philippines, Indonesia, and Vietnam etc.) can range widely from 20 to 75%, depending on the country, type of cancer, and length of follow-up [7–11]. Although additional research regarding childhood cancer treatment in Asia is unquestionably necessary, efforts should also target the emerging population of survivors.

Cancer survivorship is accompanied by the burden of a myriad of treatment-related complications that can persist for years after treatment completion. A large cohort study of adult survivors of childhood cancer in the USA revealed that at 45 years of age, the subjects had an estimated cumulative prevalence of a serious/disabling or life-threatening chronic condition of 80.5% [12]. The British Childhood Cancer Survivor Study also reported that among survivors aged at least 60 years, 31% and 37% of excess deaths were attributed to subsequent primary neoplasms and chronic cardiovascular conditions, respectively [13]. Substantial evidence in the literature indicates that these chronic morbidities can worsen the emotional health, psychosocial adjustment, and health-related quality of life of survivors of childhood cancer [14–16].

The screening and treatment of late effects in survivors of childhood cancer remain an under-researched area in most regions of Asia, despite gaining recognition as an integral component of the cancer care continuum within North America, Europe, and Oceania. Accordingly, most available data was focused on the health outcomes of survivors in Western countries, with a paucity of clinical data from Asia populations. There is a pressing need to quantify the overall risks of chronic adverse health outcomes in an emerging population of Asian survivors of childhood cancer, as this information will directly affect the allocation of resources and delivery of follow-up care services. These data may also facilitate benchmarking exercises that could eventually direct future research and service planning. This review aims to summarize the existing evidence regarding the clinical ascertainment of treatment-related complications and detection of secondary malignant neoplasms (SMN) in Asian survivors of childhood cancer. It also discusses the rationales behind the urgent need for cancer survivorship research within Asia and directions for future research.

## Methods

**Fig. 1:**
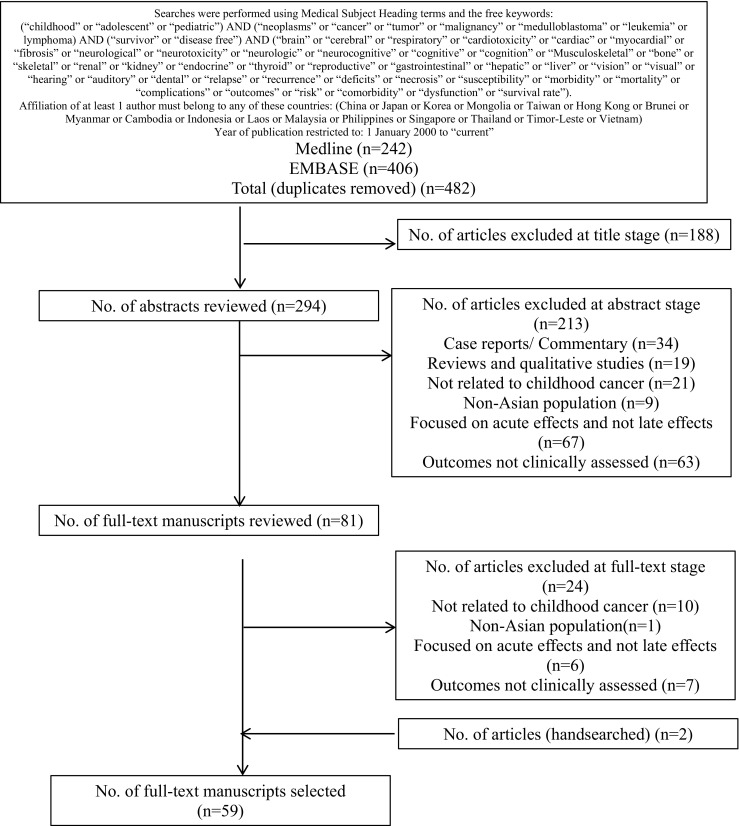
Flowchart of literature search

A search was conducted on Ovid MEDLINE and EMBASE. Inclusion and exclusion criteria were established to select studies that (1) focused on survivors of childhood cancer, defined as those who were diagnosed with cancer before the age of 19 years and had completed treatment during the time of assessment for complications; (2) used objective clinical assessments to evaluate the endpoints of SMN or/and organ-specific toxicities (cardiovascular, pulmonary, musculoskeletal, neurologic, neurocognitive, endocrine/metabolic, fertility, hearing, vision, renal, hepatic, gastrointestinal, hematological, and immunological); and (3) were published in English. In addition, studies must have been conducted on a population of childhood cancer survivors in countries or regions classified as “East Asia” (Mainland China [includes Hong Kong and Macau], Japan, Mongolia, South Korea, and Taiwan), or “Southeast Asia” (Brunei, Myanmar, Cambodia, Indonesia, Laos, Malaysia, the Philippines, Singapore, Thailand, Timor-Leste and Vietnam) by major international health and childhood cancer organizations, and consensus from the investigators [17–20]. Due to intrinsic cultural, social, and economic differences among countries across the vast continent of Asia, we did not include countries of the Middle East, South Asia, and Central Asia in the search. In light of the historical development of childhood cancer treatment protocols, this review only includes studies published during or after the year 2000 in order to provide a current perspective on the complications associated with contemporary treatment protocols for childhood cancer. The specific search terms and exclusion criteria are presented in Fig. 1. We also conducted an additional manual search of the references in the manuscripts retrieved.

We did not strictly define the term “survivor” by the duration of time post-diagnosis or post-treatment because of a lack of consensus in the literature and the use of varying criteria in international guidelines. However, studies were excluded if the treatment-related complications were assessed while patients were undergoing active therapy.

Studies with outcomes that were self- or proxy-reported without objective clinical assessments (e.g., quality of life measures and psychosocial measures) were excluded. We included only studies reporting cancer-related toxicities that had been systematically ascertained through formal clinical assessments, rather than including a combination of self- or proxy-reports of the survivors’ outcomes as discrepancies among patient self-and proxy-reports, medical records, and findings on clinical examinations are often observed [12, 21]. Meta-analyses, reviews, commentaries, or case reports comprising five or fewer cases were excluded, as were studies that did not describe the basic quantitative research methodology such as data collection methods, clinical assessment methods, and definitions and analytic and/or reporting strategies.

The search results were reviewed on three sequential levels independently by two investigators (LHP and YTC) and resolved through consensus. (1) In the initial “title stage,” the article titles were screened to exclude studies that were clearly unrelated to the main interests of this review. (2) In the “abstract stage,” the abstracts of articles that passed the “title stage” were reviewed. (3) In the final “full-text stage,” the remaining articles were examined to ensure that they fulfilled the inclusion/exclusion criteria. After that, the investigators (LHP, LP, YTC) then extracted data independently, peer-reviewed each other’s summary of the study results, and reconciled any disparities through consensus. The characteristics of the studies were systematically abstracted using a standard methodology with the following parameters: the country in which the study was conducted, publication year, study design, sample size, patient characteristics, and assessment outcomes. Lastly, the quality of each included study was assessed independently using the Quality Assessment Tool for Observational Cohort and Cross-Sectional Studies (LP and YTC) [22]. This tool was chosen as the majority of the reports are epidemiology studies which are either observational cohort studies or cross-sectional studies. Inter-rater reliability was calculated using Cohen’s kappa statistic to ascertain the agreement between each criterion of the assessment tool as scored by the reviewers [23].

## Results

The results of the literature search are depicted in Fig. 1. The search identified 482 studies from the two databases, of which 188 were excluded at the “title stage.” A total of 213 abstracts were reviewed, and 81 full-text manuscripts were subsequently appraised according to the inclusion and exclusion criteria. Finally, the systematic search yielded 59 articles that were included in this review.

### General characteristics of studies

The general characteristics (publication year, country, study design, sample size, cancer diagnoses, treatment modalities, and targeted outcomes) of the 59 studies are summarized in Supplement 1 [24–82]. A total of 13,442 subjects were represented by the included studies. The majority of the studies were conducted in the following developed countries (*n* = 52, 88%): Japan [27, 29, 30, 33, 35, 38–40, 42, 43, 45, 49, 54, 57, 59, 61, 63, 64, 67–69, 78, 80] (*n* = 24, 41%), South Korea [24, 26, 31, 32, 34, 37, 46, 53, 55, 56, 58, 60, 62] (*n* = 13, 22%), Hong Kong [41, 50–52, 72–75, 76, 77, 79, 81, 82] (*n* = 12, 20%), Taiwan [47, 48] (*n* = 2, 3%), Singapore [36] (*n* = 1, 1.5%). Only seven studies (12%) were conducted in developing countries [25, 28, 44, 65, 66, 70, 75] (Thailand and Malaysia). Half of the studies focused on either survivors of childhood leukemia [25, 27, 28, 40, 44, 46, 48, 49, 58, 63, 66, 70, 71, 73, 74, 77–79, 81, 82] (*n* = 20, 34%) or CNS tumors [43, 45, 47, 51, 52, 60, 65, 67] (*n* = 8, 13.5%), while the remainder included study samples with heterogeneous cancer diagnoses [24, 26, 29–38, 41, 50, 53–57, 59, 61, 62, 64, 68, 69, 72, 75, 76, 80] (*n* = 29, 49%) The majority of the included studies had sample sizes of 30–150 subjects (*n* = 31, 52.5%), with the exception of six studies with larger cohorts (> 500 subjects) and seven studies with less than 20 subjects. Most studies reported the survivors’ mean duration of follow-up, which ranged from 5 to 15 years after the completion of cancer therapy.

The vast majority of studies were epidemiological and explicit about the main objectives of reporting the prevalence and risk factors of the following organ-specific toxicities: cardiac [72–80] (*n* = 9, 15%); endocrine, fertility, and metabolic [53–71] (*n* = 19, 32%); neurologic [43–45, 49, 51, 52, 81, 82] and neurocognitive [46–48, 50] (*n* = 12, 20%) and SMN [36–42] (*n* = 7, 12%). Clinical endpoints such as dental [33–35], hepatic [31, 32], renal [30], and immunological [28, 29] outcomes were less commonly reported (combined *n* = 8, 14%). Four studies (7%) reported the clinical endpoints of multiple organ systems [24–27].

The assessment of study quality received an inter-rater agreement *k* of 0.82. Most studies received a “moderate” (*n* = 34, 57.6%) or “high” (*n* = 17, 28.8%) rating (Supplement 2). Due to the criteria stipulated in the literature search framework, all the included studies fulfilled certain recommended qualities of a robust epidemiological study, such as having a clearly stated objective, a specified and defined study population, cancer and its treatment preceding the assessment of late effects, and clearly defined study endpoints that were implemented consistently across study subjects. When considering the individual components of quality, most studies were considered “weak” if they did not consider varying amount of exposure (e.g., dose intensity) or did not adjust statistically for the impact of key potential confounding variables on the association between the treatment and study outcome. Sample size justification is generally not applicable to most studies that are descriptive in nature. A minority of studies (*n* = 4) with radiological assessments as endpoints reported assessments of outcomes blinded to the appropriate members of the research team.

### Cardiac outcomes

Nine studies evaluated cardiac outcomes among Chinese and Japanese survivors of childhood cancer who had been treated with anthracyclines (Table 1) [72–80]. Studies of mixed outcomes (Supplement 1), which included cardiac arrhythmia, cardiomyopathy, congestive heart failure, hypertension, hypotension, and ventricular dysfunction, reported the prevalence of cardiovascular morbidities to be between 3.5 and 16% [24–27]. Most of these studies had adopted comprehensive and advanced assessments of cardiac function, such as three-dimensional and speckle tracking echocardiography, as well as biomarkers of cardiac injury. Cheung et al. and Li et al. highlighted the presence of subclinical anthracycline-induced cardiotoxicity among asymptomatic childhood cancer survivors with normal ejection fractions [72, 73, 77, 79]. At an average of 15 years after treatment with anthracyclines, the survivors exhibited lower global longitudinal and global radial strain values relative to non-cancer controls, which suggests increased myocardial stiffness and impaired ventricular function in the former group [77]. These observations are consistent with studies conducted in Western populations using similar cardiac assessment techniques [83–85]. Authors speculated the prognostic value of incorporating myocardial strain imaging into long-term follow-up screening guidelines to detect early subclinical cardiotoxicity. These collective evidence support continued periodic cardiac surveillance among adult cancer survivors as an essential component of long-term care.

**Table 1:** Studies with cardiac outcomes

Author	Country	*N*	Sex (% male)	Diagnoses^a^	Age (Dx)	Age (follow-up)	Follow-up time	Treatment modality			Outcome assessments	Prevalence/result	Risk factors
								Chemo	Rad	HCST			
Hamada [80]	Japan	26	73%	Mixed	NR	13.5 [6–22]	5.6 [1.2–10.7] post-tx	✓ (NR)	NR	NR	Dobutamine stress ECG	• Group who received higher dose of anthracyclines had lower cardiac function at rest than other groups with lower doses. • Subclinical cardiotoxicity found even in groups with ≥ 200 mg/m^2^	• Anthracycline cumulative dose ≥ 200 mg/m^2^
Cheung [79]	HK	36	54%	Leukemia	NR	15.6 ± 5.5	7.0 [3.1–24.3] post-tx	✓ (100%)	NR	NR	Left ventricular twisting and untwisting motion	• Impairment of LV twisting NR and untwisting motion evident even in those with normal LV ejection	
Shimomura [78]	Japan	61	49%	Leukemia	5.7 ± 3.5	14.7 ± 3.5 [7.6–25.7]	8.1 [1.7–12.5] post-tx	✓ (100%)	✓ (NR)	NR	ECG, echocardiogram, serum BNP	• Ventricular premature contraction (3.3%) • Reduced exercise tolerance (12.2%) • Abnormal BNP levels (10%)	• Pirarubicin dose ≥ 300 mg/m^2^
Cheung [77]	HK	100	57%	Leukemia	8.0 [3–13]	24.1 ± 4.2	15.3 ± 5.8 post-tx	✓ (100%)	✓ (13%)	✓ (15%)	Plasma high sensitivity troponin T, conventional, 3D and speckle tracking echocardiogram	• Elevated troponin T (19%) • Worse LV myocardial deformation in survivors than controls	• Cumulative anthracycline dose • Cardiac radiation • Leukemic relapse • Stem cell transplant *• CYBA* rs4673 polymorphism
Yu [76]	HK	32	66%	Mixed	NR	19.3 ± 5.4	6.9 [2.2–14.4] post-tx	✓ (100%)	NR	NR	3D and 2D speckle tracking echocardiogram	• Impairment of subendocardial circumferential deformation and apical rotation in survivors than controls.	• Cumulative anthracyclines dose
Yu [75]	HK	53	70%	Mixed	NR	18.6 ± 5.1	7.2 [2.4–16.4] post-tx	✓ (100%)	NR	NR	3D speckle tracking echocardiogram	• Lower LV global 3D strain, twist and torsion, and LV regional deformation in survivors than controls	• Cumulative anthracyclines dose
Cheung [74]	HK	58	57%	Leukemia	7.6 ± 4.7	24.5 ± 4.4	16.6 ± 5.8 post-tx	✓ (100%)	✓ (14%)	✓ (14%)	Cardiac MRI, Tissue Doppler Imaging	• Subnormal LV ejection fraction (9%) • Abnormal and subnormal RV ejection fraction in 12% and 34%, respectively • LV fibrosis (9%) • RV fibrosis (38%)	• Cumulative anthracyclines dose
Li [73]	HK	94	56%	Leukemia	12.9 ± 6.8	22.2 ± 5.5	14.9 ± 6.2	✓ (100%)	✓ (12%)	✓ (12%)	Calibrated integrated backscatter, M-mode, Doppler and speckle tracking echocardiography	• Lower LV diastolic wall strain and stiffer LV myocardium in survivors than controls	• Older age at follow-up
Li [72]	HK	49	53%	Mixed	8.1 ± 4.5	22.9 ± 5.8	14.2 ± 5.4 post-tx	✓ (100%)	✓ (2%)	✓ (2%)	Calibrated integrated backscatter, M-mode, Doppler and speckle tracking echocardiography, plasma proANP	• Left atrial remodeling as characterized by contractile dysfunction and increased fibrosis in survivors than controls.	NR

^a^Breakdown of cancer diagnoses are presented in Table 1

Studies are arranged in chronological order

*BNP* brain natriuretic peptide, *Dx* diagnosis, *ECG* electrocardiography, *HK* Hong Kong, *LV* left ventricular, *MRI* magnetic resonance imaging, *NR* not reported, *proANP* pro-atrial natriuretic peptide, *RV* right ventricular, *tx* treatment

Interestingly, one study observed significantly higher plasma high-sensitivity cardiac troponin T levels in subjects with the CT/TT genotype at the *CYBA* gene, which plays a role in physiological free radical metabolism, compared to those with CC genotype [77]. Authors noted that the genotype distributions for this gene differed in the sample of Chinese controls (proportion with variant allele = 21.1%), compared to Caucasians (54.1%) [77, 86]. This study provided preliminary evidence that the severity of anthracycline-induced chronic cardiotoxicity may differ across ethnic groups because of genetic variants related to free radical metabolism.

### Endocrine, metabolic, and fertility outcomes

Cancer and its treatments are generally associated with a broad range of complications in the endocrine system that place the survivors of certain childhood cancers at significant long-term risks of obesity, infertility, and metabolic diseases [87].

Most included studies reported a high frequency of disorders affecting hypothalamic–pituitary–adrenal axis, thyroid, and gonadal function, especially among survivors who had been exposed to alkylating agents and/or radiation directed at these organs (Table 2). Deficiencies in growth, thyroid, and follicle-stimulating hormone levels were also detected in survivors [53, 55, 59, 60, 63, 68, 69], and a study of 122 Japanese survivors reported that half experienced gonadal dysfunction [68]. Azospermia and oligospermia were observed in 37.5% and 12.5% of male survivors treated with alkylating agents, while a quarter of female survivors required sex hormone replacement [53]. Abnormal levels of sex hormones and gonadal dysfunction (hypogonadism and decreased anti Mullerian hormone, follicle-stimulating hormone, and testosterone) were observed in 34% to 87% of participants represented by the studies.

**Table 2:** Studies with endocrine, metabolic, growth, and fertility outcomes

Author	Country	*N*	Sex (% male)	Diagnoses^a^	Age (Dx)	Age (follow-up)	Follow-up time	Treatment modality			Outcome assessments	Prevalence/result	Risk factors
								Chemo	Rad	HCST			
Yamashita [71]	Japan	21	71%	Leukemia	[1.3–14.6]	10.5–22.9	[1.3–12.5] post-tx	✓ (100%)	✓ (100%)	✓ (24%)	Linear growth, endocrinological analysis, BMD and metabolic bone markers	• Growth at post-treatment was negatively correlated with changes in height Z scores during therapy in pubertal survivors who had received chemotherapy and cranial radiation. • L2-L4 BMD less than the mean (81%)	• Changes in height Z scores during therapy
Jaruratanas-irikul [70]	Thailand	85	60%	Leukemia	5.8 ± 3.6	NR	Up to 6 years post-tx	✓ (43.5%)	✓ (37.6%)	NR	Auxological data	• Significant decrease of height trajectory, resulting in a reduction of final height of about one standard deviation or 5 cm from their genetic potential.	• Male sex (decreased height) • Female sex (overweight)
Ishiguro [69]	Japan	30	100%	Mixed	10.5 [0.9–15.8] at BMT	21.9 [15.8–29.6]	13.3 [7.6–21.2] post-BMT	✓ (100%)	✓ (83.3%)	✓ (100%)	Pubertal development, testicular Leydig cell function and germinal epithelium damage	• Puberty started spontaneously in all (100%) patients. • Normal testosterone levels but elevated luteinizing hormone level (indicating partial Leydig cell dysfunction) in 87% • One survivor (3%) fathered a child after reaching spontaneous puberty.	• Radiation without gonadal shield
Miyoshi [68]	Japan	122	51%	Mixed	6.4 [0–15]	17.3 [4–36]	8.8 [2–30] post-tx	✓ (95%)	✓ (59%)	✓ (53%)	Anthropometric measurements, BMD, hormone assays	• Endocrine abnormalities detected in 67% • Gonadal dysfunction (49%) • Growth retardation (32%) • Thyroid dysfunction (21%) • Obesity (16%) • Leanness (8%) • Central diabetes insipidus (9%) • Adrenocortical dysfunction (7%) • Low BMD (42%) • Osteoporosis (11%)	NR
Adachi [67]	Japan	23	48%	CNS tumor	NR (indicated “childhood cancer survivors”)	14.1 [4.7–22.8]	NR	✓ (NR)	✓ (NR)	✓ (4.34%) Autologous peripheral stem cell transplant	Anthropometric measurements, lipid profile	• BMI above 90th percentile (52%) • Hypercholesterolemia (17%) • Elevated fasting triglycerides (30%) • Hypoadiponectinemia (61%)	• Higher BMI
Surapolchai [66]	Thailand	131	59%	Leukemia	4 [1–15]	10 [4–20]	1.8 [0.6–6.9] post-tx	✓ (100%)	✓ (5%)	No	Anthropometric measurements, oral glucose-tolerance test, genotyping	• Impaired glucose tolerance detected in 7.6% and persisted one year after initial tests • Insulin resistance (31%)	• Older age at screening, obesity at follow-up *• PAX4* variant (R129H)
Lusawat [65]	Thailand	19	74%	CNS tumor	9.9 ± 4.6 [2.3–14.9]	[8.5–21.1]	5.8 ± 2.2 post-dx	✓ (32%)	✓ (84.2%)	No	Anthropometric measurements, GH stimulation test, ACTH stimulation test, thyroid function test	• Low peak GH (74%) • Cortisol deficiency (35%) • Central hypothyroidism (53%) • Delayed puberty (42%)	• Brain tumor location with direct HP axis involvement
Tomita [64]	Japan	51	59%	Mixed	10.5 [0.9–15.9] at HSCT	26.6 [19.4–34.3]	15.0 [6.7–27.7] post-HSCT	✓ (100%)	✓ (90%)	✓ (100%)	Anthropometric measurements, glucose and lipid metabolism profiles, abdominal CT and ultrasound, endocrine function, hormones assay	• Obesity (4%) • Underweight (male 30%; female 71%) • Fatty liver (male 37%; female 48%)	• Received cranial radiation before HSCT
Nishi [63]	Japan	6	33%	Leukemia	5 [2.7–10.2]	29.5 [21–40]	22.4 [15.5–33.9] post-dx	✓ (100%)	✓ (100%)	✓ (50%)	MRI of pituitary gland, endocrinological panel	• Hypogonadism (66.7%) • Primary hypothyroidism (16.7%)	NR
Sohn [62]	Korea	98	62%	Mixed	5.9 ± 4.9	11.2 ± 4.9	5.3 ± 2.9 post-dx 3.9 ± 2.6 post-tx	✓ (100%)	✓ (64%)	✓ (63.3%)	Anthropometric measurements; GH stimulation test, glucose and lipid metabolism profiles	• Overweight or obese (17%) • Metabolic syndrome (19%). • Median body fat percentage was 31.5% • At least one abnormal lipid value (62%) • Hypercholesterolemia (21%) • Hypertriglyceridemia (58%) • Hypertension (27%)	• Cranial radiation
Hyodo [61]	Japan	34	100%	Mixed	10.0 [0.7–15.8] at HSCT	25.1 [18.0–36.0]	16.3 [6.7–27.7] post-tx	✓ (100%)	✓ (100%)	✓ (100%)	Anthropometric measures, liver ultrasound, glucose and lipid metabolism profiles, hormones assay	• BMI < 18.5 kg/m^2^ in 32% • Fatty liver in 44% Patients who received cranial radiation therapy before SCT were more likely to develop fatty liver and insulin resistance.	• Cranial radiation
Kang [60]	Korea	28	46%	CNS tumor (Germ cell)	11.5 ± 2.4	23.1 ± 4.4	11.6 ± 5.0 post-dx 10.9 ± 5.2 post-tx	✓ (67.9%)	✓ (96.4%)	No	DEXA, anthropometric measurements, calcium, phosphate, alkaline phosphate activity, sex hormones assay	• Osteoporosis and osteopenia detected in 25% and 42.9%, respectively. • Deficiencies in growth hormone (82%), gonadotrophic hormone (68%), adrenocorticotropic hormone (64%), thyroid hormone (75%), and antidiuretic hormone (68%)	• Lower BMI • Later starting age of adult growth hormone replacement • Male sex • Low lean mass
Miyoshi [59]	Japan	53	0%	Mixed	6.3 [0–12.9]	17.4 [4.0–29.6]	8.8 [2.3–26.1] post-tx	✓ (100%)	✓ (57%)	✓ (43%)	Anti-Mullerian hormone assay, FSH assay, pubertal development	• Decreased anti-Mullerian hormone level (53%) • Increased FSH level (30%) • Abnormal breast development (17%) •No spontaneous menstruation (26%)	• Total body irradiation • Spinal radiation • Radiation of pelvis or its vicinity
Choi [58]	Korea	78	44%	Leukemia	Male: 7.2 ± 3.8 Female: 7.7 ± 3.9	Male: 11.6 ± 3.4 Female: 13.0 ± 3.3	Male: 4.4 ± 2.5 post-dx Female: 5.4 ± 3.2 post-dx	✓ (100%)	✓ (62%)	✓ (64%)	DEXA, hormones assay, anthropometric measurements	• Lumbar BMD standard deviation scores less than −2 (74%)	• Longer duration of glucocorticoid treatment for GVHD • HSCT • Chronic GVHD • Reduced BMI
Kojima [57]	Japan	49	55.1%	Mixed	5.1 [0.2–14.2]	10.7 [6.0–25.3]	5.1 [3.0–14.6] post-tx	✓ (100%)	✓ (22.5%)	✓ (32.7%)	Anthropometric measures, glucose and lipid metabolism profiles	• Metabolic syndrome in 6%. At least one and more than two components of metabolic syndrome in 37% and 20%, respectively • Hypertriglyceridemia (57%) • Hypertension (54%) • High fasting blood sugar (18%)	• Female sex
Han [56]	Korea	108	67%	Mixed	8.9 ± 4.7	20.3 ± 3.0	9.2 ± 5.2 post-tx	✓ (98%)	✓ (56%)	✓ (17%)	DEXA	• Severe BMD deficits (16%) • Moderate BMD deficits in at least one bone region (36%)	• Endocrine dysfunction • Shorter duration after treatment completion
Lee [55]	Korea	92 out of 423 (overall cohort)	66%	Mixed	4.0 [1.8–8.1]	14.4 [10.8–19.2]	4.0 [2.2–5.8] post-tx	✓ (99%)	✓ (52%)	✓ (47%)	Thyroid function	• Subclinical hypothyroidism in 24.6% of the overall cohort • Among survivors with subclinical hypothyroidism, 34% had persistent subclinical hypothyroidism	• Radiation treatment to head > 1800 cGy • Radiation to neck and spine • Lymphoma • Brain/ nasopharyngeal tumor
Adachi [54]	Japan	65	45%	Mixed	4.8 [1.0–14.3] at HSCT	15.3 ± 5.1 [6.6–27.9]	With Lipodystrophy: 18.3 [10.8–24.6] post-HSCT Without Lipodystrophy: 8.2 [3.3–26.2] post-HSCT	✓ (NR)	✓ (85%)	✓ (100%)	Liver ultrasound or CT	• Partial lipodystrophy and fatty liver disease in 9.2%, of which half of them had overt diabetes	• Older age • Longer elapsed time following HSCT • Recurrence of underlying malignant disease • History of multiple HSCT • Total body irradiation
Yoon [53]	Korea	105	54%	Mixed	13.3 [0.9–22.6]	19.7 [15.0–26.5]	6.5 [2.2–22.9] post-dx	✓ (100%)	✓ (37%)	✓ (14%)	Anti-Mullerian hormone assay, FSH assay	• Sex hormone replacement required in 27.1% of female survivors • Decreased Anti-Mullerian hormone level in 51% of female survivors • Hypogonadism (decreased testosterone) in 8.8% of male survivors • Azoospermia and oligospermia in 37.5% and 12.5% of male survivors, respectively.	• High cyclophosphamide equivalent dose in male survivors

^a^Breakdown of cancer diagnoses are presented in Table 1

Studies are arranged in chronological order

*ACTH* adrenocorticotropic hormone, *BMD* bone mineral density, *BMI* body mass index, *BMT* bone marrow transplant, *CNS* central nervous system, *CT* computed tomography, *DEXA* dual-energy X-ray absorptiometry, *Dx* diagnosis, *FSH follicle-stimulating hormone*, *GVHD* graft versus host disease, *HP* hypothalamic–pituitary, *HSCT* hematopoietic stem cell transplantation, *MRI* magnetic resonance imaging, *NR* not reported, *PNET* primitive neuroectodermal tumor, *RT* radiotherapy, *tx* treatment, *VIPN* vincristine-induced peripheral neuropathy

The prevalence of dyslipidemia (i.e., at least one abnormal lipid value, hypertriglyceridemia, or hypercholesterolemia) ranged from 50 to 60% [57, 62, 67]. Three studies evaluated the prevalence of fatty liver disease in survivors who had received cranial radiation or/and hematopoietic stem cell transplantation [54, 61, 64]. These studies found that even patients who were not overweight or obese developed fatty liver and metabolic abnormalities. Cranial radiation is most commonly associated with the risk of obesity. This association remains consistent in Asian populations, where estimates of the prevalence of obesity among Japanese and South Korean survivors range from 4 to 16% [62, 64, 67, 68], which is higher than the estimates of 3.0% to 3.2% reported in the general population of these two countries [88]. In some included studies, a low lean mass and underweight were endpoints of interest, as they were found to correlate with a lower bone mineral density and higher risk of developing osteoporosis [58, 60, 61].

### Neurologic and neurocognitive outcomes

The reported spectrum of nervous system abnormalities includes neurosensory, neurocognitive, and neurologic deficits (Table 3). The majority of the relevant studies included survivors of CNS tumors and pediatric acute lymphoblastic leukemia (ALL) who had received cranial radiation therapy [43, 45, 47, 49–52, 81, 82]. Six studies used neuroimaging to identify structural and vascular changes in the brain such as cystic malacia, cavernous angioma, moyamoya, and reduced white matter integrity [43, 45, 49–52]. The prevalence of these neuroimaging abnormalities ranged widely from 10 to 59% in survivors and was more commonly associated with CNS tumors and higher cranial radiation doses.

**Table 3:** Neurologic and neurocognitive outcomes

Author	Country	*N*	Sex (% male)	Diagnoses^a^	Age (Dx)	Age (follow-up)	Follow-up time	Treatment modality			Outcome assessments	Prevalence/result	Risk factors
								Chemo	Rad	HCST			
Chan [82]	HK	37	67.6%	Leukemia	[0.8–13]	[12–27]	[5.6–19]	✓ (100%)	✓ (100%)	No	Brain MRI, H-MRS	• Leukoencephalopathy (10.8%), infarct (2.7%), hemosiderin (59.4%) • Lower Cho/Cr and NAA/Cr observed in brains with hemosiderin	NA
Khong [52]	HK	9	NR	CNS tumor	7.8 [3–14] at tx	10.8 [3–19]	3.6 [1–6] post-tx	✓ (100%)	✓ (100%)	No	Brain MRI with DTI	• White matter at posterior fossa and supratentorial were reduced by 14.6% and 18.4%, respectively, as compared to controls.	• Younger age at treatment (< 5 years) • Longer interval since treatment (> 5 years)
Khong [51]	HK	20	70%	CNS tumor	8.6 ± 4.2 [2.9–17.4]	11.0 ± 4.6 [5.2–18.6]	2.4 [0.2–5.8] post-tx	✓ (100%)	✓ (100%)	No	Brain MRI with DTI	• Correlations found between white matter integrity and age at cranialspinal radiation and dose	• Younger age at radiation
Chan [81]	HK	64	65%	Leukemia and other solid extracranial neoplasms (mixed)	ALL: 5.2 ± 2.9 [1.2–13.7] Others: 5.9 ± 4.0 [0.5–13.0]	ALL: 17.4 ± 4.6 [6.9–27.6] Others: 15.4 ± 5.5 [7.2–31.8]	ALL: 12.2 ± 3.6 [5.0–18.8] Others: 9.5 ± 4.2 [5.6–20.4]	✓ (100%)	✓ (95.2% of ALL)	No	Brain MRI	• 62 lesions consistent with old hemorrhages in 55% of ALL patients • White matter abnormalities (4.8%) • Old infarcts (10.0%)	• Radiation dose • Time since diagnosis
Khong [50]	HK	30	66.7%	Mixed	ALL without RT: 6.68 ± 6.32 ALL with RT: 6.47 ± 4.35 CNS tumor: 8.52 ± 3.57	13.1 [6–22.1]	ALL without RT: 6.38 ± 4.29 ALL with RT: 8.39 ± 4.74 CNS tumor: 3.25 ± 2.26	✓ (100%)	✓ (70%)	NR	Brain MRI with DTI, Neurocognitive tests	• Impaired overall (17%), verbal (10%) and perceptual (20%) IQ • Impaired performance on at least one IQ subtest (53%)	• Radiation dose • Younger age at treatment
Akira [49]	Japan	6 out of 1846 (overall cohort)	66%	Leukemia	[1–15] for the overall cohort [2.1–14.1] for survivors with moyamoya	[3.2–20.9] for survivors with moyamoya	8.7 years post-dx for the overall cohort [1.5–6.8] post-dx for survivors with moyamoya	✓ (100%)	✓ (100%)	NR	Brain CT, MRI and cerebral angiography	• Cumulative incidence of moyamoya was 0.46% ± 0.02% at 8 years post-dx	• Cranial radiation
Chiou [48]	Taiwan	32	53%	Leukemia	4.4 ± 2.2 [0.8–10.8]	13.2 ± 2.5 [8.9–18.9]	8.74 ± 2.3 [5.3–13.9] post-dx	✓ (100%)	✓ (19%)	No	Neurocognitive tests for IQ, memory, executive function, visual spatial, attention, information processing speed and motor skills	• Impaired IQ (15.6%) • Impairment in one or more cognitive domains (27.8%)	NR
Liang [47]	Taiwan	56	77%	CNS tumor (germ cell)	11.9 [3.2–19.9]	17.7 [8.9–29.1]	6.9 [1.7–17.9] post-tx	✓ (48%)	✓ (96%)	No	Neurocognitive tests for IQ, memory, verbal and visual constructional memory, attention, executive function and visual organization	• Patients with tumors in the basal ganglia region had lower IQ than those with tumors in the pineal or suprasellar regions.	• Tumors in the basal ganglia region • Extensive irradiation field • High irradiation dosage
Kim [46]	Korea	42	60%	Leukemia	3.8 ± 2.3	10.5 ± 2.4	6.6 ± 1.3 post-dx	✓ (100%)	✓ (43%)	No	Neurocognitive tests for IQ, executive function and attention	• Lower but non-significant IQ in survivors than healthy controls • Worse attention and executive function in survivors than healthy controls.	• Cranial radiation • Male • Younger age at diagnosis
Yamasaki [45]	Japan	25	52%	CNS tumor	[2.3–15.8]	NR	7.5 [1.3–24.2] post-dx	✓ (NR)	✓ (100%)	No	Brain MRI	• Multiple cavernous angioma (52%)	• Radiation therapy at age younger than 6 years • PNET • Pineoblastoma
Tay [44]	Malaysia	101	66%	Leukemia	5.3 ± 3.2 [0.4–12.9]	11.8 ± 3.8 [4.8–18.0]	4.1 ± 2.1 [2.0–10.2] post-tx	✓ (100%)	No	NR	Electrophysiological nerve conduction studies, gross and fine motor function, VIPN	• Both clinical and electrophysiological neuropathy abnormalities (15.8%)	• Intermediate or high-risk stratification treatment arms
Yamasaki [43]	Japan	41	63%	CNS tumor	9 [3.3–15.7]	NR	7.2 [1.2–15.8] months post-dx	✓ (NR)	✓ (100%)	No	Brain MRI	• Cystic malacia detected in 26.8% at a median of 30.8 months [14.9–59.3 months] • White matter changes (46%)	• Younger age at radiation • Supratentorial location of tumors

^a^Breakdown of cancer diagnoses are presented in Table 1

Studies are arranged in chronological order

*ALL* acute lymphoblastic leukemia, *Cho/Cr* choline/creatine ratio, *CNS* central nervous system, *CT* computed tomography, *DTI* diffusion tensor imaging, *Dx* diagnosis, *HK* Hong Kong, ^*1*^*H-MRS* proton magnetic resonance spectroscopy, *IQ* intelligence quotient, *MRI* magnetic resonance imaging, *NAA/Cr* N-acetylaspartate/creatine ratio, *NR* not reported, *PNET* primitive neuroectodermal tumor, *RT* radiotherapy, *tx* treatment, *VIPN* vincristine-induced peripheral neuropathy

Neurocognitive studies identified impairment in the overall and perceptual intelligence in 10% to 28% survivors of CNS tumor and leukemia [47, 48, 50]. One study found that half of the survivors demonstrated impaired performance on at least one of the IQ subtests [50]. Few studies have evaluated the neurocognitive outcomes of survivors of other childhood cancers not typically treated with neurotoxic therapies, including neuroblastoma, germ cell tumor, Hodgkin lymphoma, and retinoblastoma.

Whereas most studies focused on CNS toxicities, Tay et al. evaluated the prevalence of vincristine-inducted peripheral neuropathy in survivors of childhood ALL at 2 years post-treatment [44]. The authors found that 15.8% of the survivors exhibited combined clinical and electrophysiological neuropathy, which was associated with poorer physical functioning [44].

### Secondary malignant neoplasms

The development of SMN is a well-established sequela among long-term survivors of childhood cancer. Among subgroups of survivors, the risk of SMN is related to previous treatment with ionizing radiation, hematopoietic stem cell transplantation, and specific chemotherapies.

All the included studies used in-house medical databases or registries in their countries to identify cases of SMN in survivors of childhood cancer (Table 4) [36–42]. The incidence of SMN remained low even though the risk of secondary cancer remains 6 to 12 times higher than that in the general population. Estimated SMN prevalence rates differ among cohorts of Asian and Western survivors because of differences in the epidemiology of primary cancer diagnoses, treatment strategies, and genetic factors. Interestingly, there are also slight differences in the 20-year cumulative incidences of SMN across Japan [38] (3.2%), Hong Kong [41] (2.9%), and Singapore [36] (5.3%), though these differences may not be clinically relevant and may likely be attributable to the treatment era and exposure to different types and intensities of treatments.

**Table 4:** Studies with secondary malignancy outcomes

Author	Country	*N*	Sex (% male)	Diagnoses^a^	Age (Dx)	Age (follow-up)	Follow-up time	Treatment modality			Outcome assessments	Prevalence/result	Risk factors
								Chemo	Rad	HCST			
Araki [42]	Japan	744	51%	Retinoblastoma	< 1 year: 48% ≥ 1 year: 52%	NR	SMN: 8.5 [2–36.5] post-dx No SMN: 9.1 [0–49.5] post-dx	✓ (NS)	✓ (56%)	No	Records of SMN	• Twenty-one cases (2.8%) developed 23 SMN • Most frequent SMN were osseous or soft tissue sarcomas	• Younger age at diagnosis • Hereditary • Focal therapy • Focal chemotherapy • Systemic chemotherapy • External beam irradiation
Sun [41]	HK	1233	43%	Mixed	6.3 [0–20.1]	NR	5.3 [0–26.1] post-dx	✓ (NR)	✓ (NR)	✓ (NR)	Pathological reports of suspected SMN	• Twelve cases developed SMN with 10-year and 20-year cumulative incidence of 1.3% and 2.9%, respectively. • Most frequent SMN were acute leukemia or myelodysplastic syndrome and central nervous system tumor. • Median interval between diagnosis of primary and SMN was 7.4 [2.1–13.3] years	• Radiotherapy in patients with acute lymphoblastic leukemia
Ishida [40]	Japan	1716	NR	Mixed	[1–15]	NR	Every 2 years	✓ (100%)	✓ (NR)	✓ (NR)	Records of SMN	• Thirty-seven cases of SMN (2%) • Most frequent SMN were AML, MDS, non-Hodgkin lymphoma and CNS tumors • Median latency period from ALL diagnosis to secondary • Cancers was 6 years (range 1–23 years)	• Cranial radiation, especially moderate and high doses • Age at ALL diagnosis > 7 years • Inclusion in more recent protocols
Fujiwara [39]	Japan	857	NR	Retinoblastoma	0.3 [0.1–1.7] for the 10 patients who developed SMN	NR	10.3 [7–24.1] post-dx for the 10 patients who developed SMN	✓ (NR)	✓ (NR)	No	Secondary osteosarcoma	• Ten cases (1.1%) developed second primary osteosarcoma • The latent period from diagnosis of retinoblastoma until the diagnosis of second primary osteosarcoma was 10.3 [7 to 24.1] years.	NR
Ishida [38]	Japan	5387 (5-year survivors)	57%	Mixed	5.4 ± 4.5	17.9 ± 7.1	11.2 [5.0–30.0] post-dx	✓ (91%)	✓ (40%)	✓ (55%)	Records of SMN	• Cumulative incidence of SMN is 1.2% at 10 years and 3.2% at 20 years from the time of primary cancer diagnosis	• Retinoblastoma bone/soft tissue sarcomas allogeneic SCT • Older age at primary diagnosis (> 7 years) • Attained age < 9 years
Koh [37]	Korea	102	55%	Mixed	6.6 [0–19.7]	12.7 [2.5–29.4]	8.6 [1.2–27.5]	✓ (NR)	✓ (NR)	✓ (NR)	Records of SMN	• Median interval between primary cancer diagnosis and SMN is 4.9 [0.5–18.5], with the shortest interval for AML and MDS	NR
Lim [36]	Singapore	1124	60%	Mixed	5.4 [0–20.7]	NR	3.5 [0–24.1]	✓ (NR)	✓ (NR)	✓ (NR)	Pathological reports of suspected SMN	• Fifteen cases developed SMN (1.3%) • Overall 20-year cumulative incidence of SMNs was 5.3% • Median interval between primary cancer diagnosis and SMN was 3.4 [0.2 to 18.3] years	• Topoisomerase II inhibitor • Osteosarcoma

^a^Breakdown of cancer diagnoses are presented in Table 1

Studies are arranged in chronological order

*ALL* acute lymphoblastic leukemia, *AML* acute myeloid leukemia, *Dx* diagnosis, *HK* Hong Kong, *MDS* myelodysplastic syndromes, *NR* not reported, *NS* not specific, *SCT* stem cell transplant, *SMN* second malignant neoplasm

### Other outcomes

Three studies evaluated chemotherapy-induced tooth formation anomalies in survivors with mixed cancer diagnoses (Supplement 3) [33–35]. Approximately 55 to 80% of survivors developed at least 1 oral or maxillofacial abnormality, especially those who received hematopoietic stem cell transplantation and were treated at a younger age.

Lee et al. and Yoo et al. retrospectively reviewed the clinical and imaging features of hypervascular hepatic nodules in survivors [31, 32]. Both studies concluded that these nodules are benign and unlikely to undergo a malignant change.

Two studies examined the immunological outcomes of survivors [28, 29]. Azanan et al. reported higher levels of inflammatory cytokines and T cell responses specific to *cytomegalovirus* among survivors of childhood leukemia relative to non-cancer controls, while Mahmoud et al. detected chronically elevated levels of Epstein–Barr virus DNA in 20% of the survivors [28, 29]. Additionally, Azanan and colleagues discussed the association between the development of age-related comorbidities and the immune phenotype of aging, as characterized by increased levels of circulating inflammatory cytokines, among survivors [28].

## Discussion

To our knowledge, this is the first systematic review of the available data on the chronic health outcomes among survivors of childhood cancer in Asia. Currently, the majority of survivorship research in the literature originated in North America, Europe, and Oceania, in contrast to the paucity of robust studies from Asia. Our review is unique in that we included only clinical ascertainment of health outcomes that are well-defined and were consistently applied to the populations of each study. We believe that this approach has strengthened the robustness of the review, as reflected by the moderate to high methodological quality of most included studies. Although the studies used different medical evaluation methods and clinical endpoint definitions, the collective findings summarize the existing types of chronic health problems carried by childhood cancer survivors among different ethnic groups across Asia.

Our results suggest that the status of survivorship research within Asia closely reflects the disparities of the available resources. Most survivorship studies in this review were conducted in highly developed countries such as Japan, South Korea, Hong Kong, and Singapore, which share common characteristics such as a high childhood cancer survival rate, established cohorts of childhood cancer survivors, and a high research prioritization of cancer. However, published data and statistics from national registries reveal that up to 70–80% of children with cancer reside in low- to middle-income countries that still struggle with high cancer-related mortality rates [7, 8]. Many countries in Southeastern and South Asia continue to face challenges such as poor access to timely and affordable treatment and a lack of sustainable local pediatric oncology programs [1]. Although continued progress in childhood cancer treatment remains necessary, efforts should also target survivorship care and research among the emerging population of survivors, especially in mainland China and other developing Asian countries. As the susceptibility to and pathogenesis of cancer- and treatment-related chronic health conditions are heavily influenced by genetic factors, environmental exposures, and health behaviors, research efforts must expand to include cases in developing countries.

Importantly, evidence from survivorship studies conducted in Western populations cannot be extrapolated to Asian survivors because of genetic differences in drug responses and susceptibilities to the development of adverse, chronic treatment-associated toxicities. The concept of pharmacoethnicity, which is broadly defined as both genetic and non-genetic ethnic diversity in drug responses and toxicities, is an emerging hypothesis proposed to explain inter-individual and inter-ethnic variations in drug responsiveness [89, 90]. Different rates of chemotherapy-induced toxicities among Caucasian, African American, Asian, and Hispanic cancer populations have been attributed to variations in pharmacogenetics [90–92]. These variations include inherited genetic polymorphisms that render subgroups of cancer survivors more susceptible to certain late effects. For example, one included study identified genetic variations in anthracycline-induced chronic cardiotoxicity [77], and a high-risk genetic profile for premature menopause was recently identified among childhood cancer survivors who had been exposed to gonadotoxic therapy [93]. Furthermore, inert genetic polymorphisms related to underlying diseases might determine susceptibility to the development of adverse outcomes associated with cancer treatment among survivors. The results of epidemiological studies of general populations suggest that the risk of neurocognitive deficits associated with apolipoprotein E polymorphisms differs among ethnic groups, such that stronger gene-disease associations were observed in Japanese subjects than in Caucasian subjects [94, 95]. The systematic characterization of the genetic variability in treatment-related complications among Asian survivors is expected to have important clinical implications in this new era of precision medicine.

The non-genetic aspects of cancer pharmacoethnicity include environmental factors and culturally related behaviors, which may have strong influences on drug bioavailability and metabolism [89, 91]. From a biological perspective, the effects of inflammation and oxidative stress on the development of chronic diseases and symptoms in survivors may differ between Western and Asian survivors because of differences in health habits such as physical activity, dietary patterns, and risky behaviors (e.g., alcohol consumption and tobacco use). Although heterogeneity among the study samples in terms of the cancer diagnoses and age at the time of follow-up precludes a meaningful conclusion, our review found that estimates of the prevalence of obesity (4% to 16%) among Asian survivors were considerably lower than that observed among Western survivors of childhood cancer. Specifically, the reported prevalence rates of overweight/obesity were 26% among Swiss survivors of childhood cancer and 26.2% in the St. Jude Lifetime Cohort Study (USA) [12, 96]. Studies of survivors of childhood ALL have identified associations of biomarkers of vascular injury and inflammation with treatment-induced cardiovascular conditions (hypertension, dyslipidemia, and metabolic syndrome), sleep disturbances, and fatigue [97–99]. Efforts to identify the causative carcinogenic factors and the contributions of environmental and health behavior factors to the development of SMN should maintain the efficacy of contemporary treatments provided to survivors. For example, environmental pollution is known to contribute to cancer epidemiology, the incidence and geographical distribution of SMN in China [100]. These differences might influence the trajectories of late treatment complications during the survivorship phase and should be considered in studies of chronic diseases and SMN in survivors across ethnic groups.

## Directions for future research

This review highlights the need to improve survivorship care and research efforts among Asian survivors of childhood cancer. We propose the following three research priorities:

### Opportunities to create a research repository of outcomes data within Asia

Medical information collected via the prospective systematic screening of late effects in survivors could serve as a framework for future epidemiological and interventional studies unique to the Asian population of childhood cancer survivors. Existing models of survivorship research programs in Western countries could thus be adopted and adapted to spearhead similar initiatives in Asia. One such example is the Childhood Cancer Survivorship Study (CCSS) consortium, which was formed in 1994 by a group of 31 contributing clinical pediatric centers in the USA and Canada [101]. The CCSS is composed of individuals who survived five or more years after diagnosis of childhood cancer and siblings of survivors who serve as the comparison group for the study. The initiation of such a collaborative effort will not merely facilitate the systematic surveillance of survivors and collection of clinical data across different regions of Asia, but will also help to address the research gaps outlined in this review. The collected aggregate information can be used to form a repository of outcome data that will comprise a valuable research resource readily available to investigators with different professional backgrounds (e.g., medical, nursing, pharmacy, psychology, social work, health services) who are interested in studying the late outcomes of childhood cancer survivors. Notably, multinational studies are often labor-intensive, resource-demanding and likely to involve many investigators and stakeholders, as well as the coordination of study protocols. Existing infrastructures, such as the Asian continental branch of the International Society of Pediatric Oncology (SIOP-Asia) and Childhood Cancer International (CCI), may pave the way for such regional initiatives and collaborative efforts across countries in Asia.

### Genetic markers to predict cancer outcomes and late effects

Studies on both cancer genomes and germline genomes have provided unprecedented insights into the molecular nature of cancer and inter-patient variance [102]. Mounting studies have demonstrated the superiority of machine learning and artificial intelligence in clinical diagnosis and prognosis of childhood cancer [103]. The whole landscape of understanding host and cancer genome plays a crucial role in precision medicine, which will eventually lead to individualized therapies to achieve better response while minimizing long-term toxicities.

Much effort has been initiated on this subject matter over the recent years, particularly in Asia. For example, inherited *TPMT* and *NUDT15* variants are essential genetic markers for mercaptopurine dosage adjustment [104, 105]. Notably, the *NUDT15* variant occurs more frequently in East Asians and Hispanics, as compared to Europeans and Africans [104]. Inherited *TP53* variants are significantly associated with poorer outcomes and higher secondary cancer incidence in children with ALL [106] while novel ALL subtype (Ph-like ALL) based on gene expression profiling may guide the use of targeted therapies in high-risk patients [107].

Recognizing the power of exploiting genomics data at a population level, China has launched the “100,000 Genomes Project” in 2017, the world’s largest genome project, to study the interactions among genetic inheritance, diseases, treatments, and quality of life. However, the overall funding and research involvement for childhood cancer in Asia still substantially lag behind the European and American countries. Applying genetic information to guide the development and selection of childhood cancer therapies in the clinic is promising, yet challenging and requires a multi-national effort. We propose priorities for a research agenda to first pool existing genetic association studies among the Asian cancer population to more accurately quantify the magnitude of risks and functional significance of the variants, and then harness bioinformatics technology to inform reliable prediction models for treatment-related late toxicities. Secondly, research is needed to develop evidence-based guidelines concerning genetic susceptibility testing for late toxicities and implementing them into practice. It is hoped that the current trend toward specialized laboratories, genetic reporting services, and personnel within the Asian region will allow researchers to access gene expression and digitized phenotypic data of patients across national borders.

### Novel culturally relevant strategies to prevent or/and treat late effects

Complementary and alternative medicine (CAM) is broadly defined as medical products and practices that are not part of standard clinical care. Within this broad spectrum of modalities is Traditional Chinese Medicine (TCM). Though the prevalence estimates of CAM use were often evaluated in children with cancer during the active treatment phase [108, 109], it can be reasonable to infer that CAM use may be as prevalent during the survivorship phase, given that TCM is one of the predominating CAM approaches particularly among patients of Chinese ancestry in Asia and worldwide [110].

Despite the growing interest and popularity in CAM use among pediatric patients, the safety and effectiveness of CAM among survivors of childhood cancer are under-researched. Many of these approaches may not be evidence-based due to the difficulty of standardizing the products used and approaches or techniques employed. These impending trends underscore the need for effective models of integrative care that are both evidence-based and culturally sensitive [111, 112]. Further research work evaluating the use of CAM among survivors of childhood cancer may help clarify and answer the many questions and concerns regarding the use of complementary and conventional therapy and better delineate its role in health care needs of childhood cancer survivors.

## Implications and recommendations for clinical practice

### Toward the systematic screening of health outcomes

International working groups, such as the Children’s Oncology Group (COG), American Society of Clinical Oncology (ASCO), and International Society of Pediatric Oncology (SIOP), now support the need for evidence-based systematic screening of late effects in survivors with childhood cancer [113, 114]. For example, the “Children’s Oncology Group Long-Term Follow-Up Guidelines for Survivors of Childhood, Adolescent, and Young Adult Cancers” includes risk-based, exposure-related clinical practice guidelines that provide recommendations for the screening and management of late effects in survivors [115]. “Risk-based” or “exposure-based” care refers to a personalized, systematic plan of regular screening, surveillance, and prevention strategies based on the patient’s treatment and cancer history [114]. Such evidence-based guidelines may facilitate a recommendation for the standardized risk-based screening of asymptomatic survivors of childhood, adolescence, or young adulthood cancers who present for routine exposure-related medical follow-up visits.

In every country, existing constraints within the current clinical setting and healthcare system limit the implementation of all aspects of risk-based guidelines. For example, our review found that the neurodevelopmental trajectory is less explored in children with cancer in Asia. This is an important research gap in the literature, especially as educational attainment and academic achievement are highly regarded in most Asian societies. This may be limited by the lack of certified neuropsychologists and developmental psychologists in Asia [116–118]. Therefore, international guidelines must be adapted and modified to meet the needs of the local healthcare structure. Health policy and health system research is needed to weigh other key considerations, which include reimbursement policies for screening tests implemented by the government and private insurance companies, the availability of resources, and labor constraints in the healthcare field.

### Improving access to survivorship care

Primary pediatric clinics often lose survivors of childhood cancer to follow-up because these patients experience increased independence and mobility as they advance into adulthood. Particularly in Asia, the needs of this population are often understudied and under-addressed. Few Asian countries have long-standing programs to provide regular follow-up evaluations and manage the health needs of childhood cancer survivors, and socioeconomic disparities within this region also pose challenges to the abilities of survivors to access such services, even if they are available.

As survivors transit from pediatric to adult care, they face complex issues that require family-centered, inter-related care coordination, and gradual step-down care within the community [119, 120]. Currently, there is a clear lack of studies evaluating the specific barriers to long-term survivorship care in the pediatric oncology field. However, studies of the barriers to cancer survivorship faced by Chinese breast cancer patients revealed that survivors were wary of entrusting their complex health conditions to primary care physicians, whom they perceived to be poorly equipped to manage cancer-related health problems [121]. One study also reported that Japanese survivors of childhood cancer preferred to receive long-term care from the same physician as adults [122]. Supplement 4 summarizes the potential gaps and barriers to implementing a comprehensive survivorship program based on experiences from European and North American populations [113, 120, 123, 124], as well as experience and existing interventions in the Asian population [26, 125–133]. Future work should aim to identify effective and sustainable transition models and create recommendations for and formal evaluations of successful transitional care. These efforts should involve the development of a structured program to facilitate the safe and efficient distribution of clinical services to a multidisciplinary team of primary care providers, allied health professionals, and non-governmental organizations (NGOs). These programs should also include elements such as financial services, patient navigators, and social workers to help patients prepare for the economic challenges related to cancer and its treatment.

Over the next decade, international and regional collaborations may play an important role in improving access to survivorship care in developing countries. The concept of “twinning,” in which a cancer center from a developed country collaborates with another center in a developing country, has been used successfully to improve the childhood cancer survival rates in some countries [134]. Hopefully, such initiatives can be extended to survivorship care within the near future. The two-way transfer of survivorship-related expertise, advice, knowledge, and skills may enhance the construction of infrastructure for survivorship work in developing countries.

### Interventions to improve health literacy levels

Education and empowerment are crucial for survivors of childhood cancer as they assume age-appropriate ownership of their health and become active partners during the survivorship phase [124]. This process includes the promotion of protective health behaviors. Poor health-based knowledge of the importance of detecting treatment-related late complications during the early years of survivorship may affect their health habits during adulthood (e.g., sun protective behaviors, smoking, screening for SMN). A recent study of survivors of childhood cancer in Hong Kong described how their perceptions of fatigue and reduced physical strength after the cancer diagnosis had prevented them from being physically active [135]. Other studies have highlighted that Korean cancer survivors face challenges regarding smoking cessation, as smoking is perceived as a strategy for pain and stress relief [136, 137]. The promotion of health behavior education among survivors is a timely topic in Asia, where many health and research programs have been implemented to ameliorate the general adverse health effects of the urban environment on children and adolescents, such as sleep disturbances, sedentary lifestyles, and academic stress.

In the USA and Europe, many survivorship programs now incorporate individualized counseling on the personal health risks faced by survivors and caregivers. This includes a review of the survivors’ medical and treatment histories to facilitate collaborations among survivors, caregivers, and oncology practitioners in the development of personalized care plans [113, 123, 124]. To address the relevant therapy-related health risks, counseling also promotes culturally relevant protective health behaviors and aims to reduce harmful health behaviors. Such practices are less commonly applied in Asia, and this is complicated by a lack of patient-friendly resources on late effects available in the patients’ native languages, especially in developing Asian countries. Personalized care requires providing relevant educational resources that will allow oncology practitioners to address the health effects of treatment. Advances in technology and teleoncology programs may increase both the access to and quality of clinical cancer care through collaborative efforts among medical centers, academic institutions, and NGOs [138]. One viable method involves the use of Internet-based, mobile health, and other innovations to disseminate vital educational information to survivors in developing countries and deprived regions throughout Asia. Given the burden of diseases in these developing countries, the successful application of technology to empower patients during their transition from active treatment to survivorship may potentially have a substantial societal effect.

## Limitations

Although this review has several strengths, such as the inclusion of studies with objective assessments of clinical outcomes and clear definitions of study samples, a few limitations are worth noting. Based on the present studies, it is difficult to estimate the overall prevalence of specific late toxicities in Asia, given the heterogeneity of the study populations and the lack of standardized assessments and definitions of clinical endpoints. Several studies were based on findings from single institutions within a single country. Hence, the treatment characteristics of those study populations may be different from those conducted at other institutions. For example, one included study reported a 5-year cumulative SMN incidence after osteosarcoma of 14.1% in Singapore, which is considerably higher than the rates reported in other countries, as well as at a medical center in the USA that used a similar treatment protocol for osteosarcoma [36]. However, this single-centered data may be an overestimation of SMN incidence and the authors themselves concluded that this concerning observation warrants further studies of risk factors that might be specific to their population.

As the majority of the included studies are research-driven, there is a potential surveillance bias between the study population and the general childhood cancer population, especially for asymptomatic or subclinical conditions that may be captured by screening and in-depth clinical assessment in a research study, but not in routine clinical setting. It is likely that survivors participating in research studies were more closely assessed over time leading to an overestimation of the prevalence of late effects. Finally, our systematic review found no prevalence data for most Asian countries, including mainland China, Bangladesh and Mongolia, although this may be attributable to the inclusion of only studies and abstracts written in English, as it is methodologically difficult to conduct search and translation processes for literature written in the multiple languages represented in Asia. A formal meta-analysis was also not conducted because the above limitations would severely restrict the ability to draw meaningful conclusions.

## Conclusion

The continued development of national and international collaborative initiatives will yield steady advances in the treatment of childhood cancers throughout Asia over the next few decades. Although additional research regarding childhood cancer treatment in Asia is unquestionably necessary, efforts should also target the emerging population of survivors, particularly in terms of survivorship care. Innovative and collaborative strategies are needed to improve access to quality survivorship care, especially in mainland China and other developing countries in Asia.

Preparation for the impending demands of an emerging population of childhood cancer survivors should begin now. The collective evidence generated by this review, as well as existing literature, may contribute to emerging collaborative research throughout Asia and thus address the needs of survivors on a regional scale. Collaborative initiatives, which will be facilitated by the construction of a regional repository of systematically assessed health outcomes and biospecimens, is expected to facilitate impactful studies of treatment-related, social–environmental and genetic predictors and interventions for late effects in this population.

## Electronic supplementary material

ESM 1 (DOCX 329 kb)
