# Supplementary material for: Clinical ascertainment of health outcomes in Asian survivors of childhood cancer: a systematic review
Source: J Cancer Surviv. 2019 May 4;13(3):374–96. doi: 10.1007/s11764-019-00759-9 (PMC6548762; doi:10.1007/s11764-019-00759-9)
Supplement: Supplementary file 1 — (DOCX 329 kb) [file 11764_2019_759_MOESM1_ESM.docx]

Supplement 1: Characteristics of Included Studies

|  | Author | Year | Country | Design | Diagnoses | | | | | | | | | Total N |
| --- | --- | --- | --- | --- | --- | --- | --- | --- | --- | --- | --- | --- | --- | --- |
|  |  |  |  |  | Leukemia | CNS tumor | Lymphoma | Retinoblastoma | Germ cell | Neuroblastoma | Renal | Bone | Others |  |
| Cardiac Outcomes | | | | | | | | | | | | | | |
| 1 | Hamada[80] | 2006 | Japan | Single-centered | 24 | 0 | 2 | 0 | 0 | 0 | 0 | 0 | 0 | 26 |
| 2 | Cheung[79] | 2011 | Hong Kong | Single-centered | 36 | 0 | 0 | 0 | 0 | 0 | 0 | 0 | 0 | 36 |
| 3 | Shimomura[78] | 2011 | Japan | Multi-centered | 61 | 0 | 0 | 0 | 0 | 0 | 0 | 0 | 0 | 61 |
| 4 | Cheung[77] | 2013 | Hong Kong | Multi-centered | 100 | 0 | 0 | 0 | 0 | 0 | 0 | 0 | 0 | 100 |
| 5 | Yu[76] | 2013 | Hong Kong | Single-centered | 24 | 0 | 3 | 0 | 0 | 0 | 0 | 5 | 0 | 32 |
| 6 | Yu[75] | 2013 | China | Single-centered | 36 | 0 | 6 | 0 | 0 | 1 | 0 | 9 | 1 | 53 |
| 7 | Cheung[74] | 2015 | Hong Kong | Multi-centered | 58 | 0 | 0 | 0 | 0 | 0 | 0 | 0 | 0 | 58 |
| 8 | Li[73] | 2016 | Hong Kong | Single-centered | 94 | 0 | 0 | 0 | 0 | 0 | 0 | 0 | 0 | 94 |
| 9 | Li[72] | 2017 | Hong Kong | Single-centered | 27 | 0 | 12 | 0 | 0 | 2 | 1 | 7 | 0 | 49 |
| Endocrine, Metabolic, Growth and Fertility Outcomes | | | | | | | | | | | | | | |
| 1 | Yamashita[71] | 2003 | Japan | Single-centered | 21 | 0 | 0 | 0 | 0 | 0 | 0 | 0 | 0 | 21 |
| 2 | Jaruratanas-irikul[70] | 2004 | Thailand | Single-centered | 85 | 0 | 0 | 0 | 0 | 0 | 0 | 0 | 0 | 85 |
| 3 | Ishiguro[69] | 2007 | Japan | Single-centered | 16 | 0 | 3 | 0 | 0 | 0 | 0 | 0 | 11 | 30 |
| 4 | Miyoshi[68] | 2008 | Japan | Single-centered | 52 | 15 | 10 | 0 | 15 | 9 | 2 | 0 | 19 | 122 |
| 5 | Adachi[67] | 2009 | Japan | Single-centered | 0 | 23 | 0 | 0 | 0 | 0 | 0 | 0 | 0 | 23 |
| 6 | Surapolchai[66] | 2010 | Thailand | Single-centered | 131 | 0 | 0 | 0 | 0 | 0 | 0 | 0 | 0 | 131 |
| 7 | Lusawat[65] | 2010 | Thailand | Single-centered | 0 | 19 | 0 | 0 | 0 | 0 | 0 | 0 | 0 | 19 |
| 8 | Tomita[64] | 2011 | Japan | Single-centered | 46 | 0 | 5 | 0 | 0 | 0 | 0 | 0 | 0 | 51 |
| 9 | Nishi[63] | 2011 | Japan | Single-centered | 6 | 0 | 0 | 0 | 0 | 0 | 0 | 0 | 0 | 6 |
| 10 | Sohn[62] | 2011 | South Korea | Single-centered | 31 | 10 | 0 | 5 | 23 | 25 | 0 | 0 | 4 | 98 |
| 11 | Hyodo[61] | 2012 | Japan | Single-centered | 17 | 0 | 4 | 0 | 0 | 0 | 0 | 0 | 13 | 34 |
| 12 | Kang[60] | 2012 | South Korea | Single-centered | 0 | 28 | 0 | 0 | 0 | 0 | 0 | 0 | 0 | 28 |
| 13 | Miyoshi[59] | 2012 | Japan | Single-centered | 14 | 15 | 5 | 0 | 2 | 5 | 3 | 0 | 9 | 53 |
| 14 | Choi[58] | 2013 | South Korea | Single-centered | 78 | 0 | 0 | 0 | 0 | 0 | 0 | 0 | 0 | 78 |
| 15 | Kojima[57] | 2013 | Japan | Single-centered | 35 | 0 | 3 | 0 | 0 | 0 | 0 | 0 | 11 | 49 |
| 16 | Han[56] | 2015 | South Korea | Single-centered | 49 | 11 | 29 | 0 | 2 | 1 | 4 | 5 | 7 | 108 |
| 17 | Lee[55] | 2015 | South Korea | Single-centered | 32 | 8 | 16 | 0 | 0 | 36 | | | | 92/423 |
| 18 | Adachi[54] | 2017 | Japan | Single-centered | 31 | 8 | 2 | 0 | 0 | 16 | 0 | 3 | 5 | 65 |
| 19 | Yoon[53] | 2017 | South Korea | Single-centered | 23 | 18 | 17 | 0 | 0 | 0 | 0 | 46 | 1 | 105 |
| Neurologic and Neurocognitive Outcomes | | | | | | | | | | | | | | |
| 1 | Chan[82] | 2001 | Hong Kong | Single-centered | 37 | 0 | 0 | 0 | 0 | 0 | 0 | 0 | 0 | 37 |
| 2 | Khong[52] | 2003 | Hong Kong | Single-centered | 0 | 9 | 0 | 0 | 0 | 0 | 0 | 0 | 0 | 9 |
| 3 | Khong[51] | 2004 | Hong Kong | Single-centered | 0 | 20 | 0 | 0 | 0 | 0 | 0 | 0 | 0 | 20 |
| 4 | Chan[81] | 2006 | Hong Kong | Single-centered | 43 | 0 | 3 | 0 | 0 | 2 | 6 | 4 | 6 | 64 |
| 5 | Khong[50] | 2006 | Hong Kong | Single-centered | 18 | 12 | 0 | 0 | 0 | 0 | 0 | 0 | 0 | 30 |
| 6 | Akira[49] | 2007 | Japan | Single-centered | 6/1846 | 0 | 0 | 0 | 0 | 0 | 0 | 0 | 0 | 6/1846 |
| 7 | Chiou[48] | 2009 | Taiwan | Single-centered | 32 | 0 | 0 | 0 | 0 | 0 | 0 | 0 | 0 | 32 |
| 8 | Liang[47] | 2013 | Taiwan | Single-centered | 0 | 56 | 0 | 0 | 0 | 0 | 0 | 0 | 0 | 56 |
| 9 | Kim[46] | 2014 | South Korea | Single-centered | 42 | 0 | 0 | 0 | 0 | 0 | 0 | 0 | 0 | 42 |
| 10 | Yamasaki[45] | 2015 | Japan | Single-centered | 0 | 25 | 0 | 0 | 0 | 0 | 0 | 0 | 0 | 25 |
| 11 | Tay[44] | 2016 | Malaysia | Single-centered | 101 | 0 | 0 | 0 | 0 | 0 | 0 | 0 | 0 | 101 |
| 12 | Yamasaki[43] | 2017 | Japan | Single-centered | 0 | 41 | 0 | 0 | 0 | 0 | 0 | 0 | 0 | 41 |
| Secondary Malignancy | | | | | | | | | | | | | | |
| 1 | Araki[42] | 2011 | Japan | Single-centered | 0 | 0 | 0 | 744 | 0 | 0 | 0 | 0 | 0 | 744 |
| 2 | Sun[41] | 2011 | Hong Kong | Single-centered | 550 | 159 | 79 | 14 | 59 | 71 | 35 | 101 | 165 | 1233 |
| 3 | Ishida[40] | 2013 | Japan | Single-centered | 1716 | 0 | 0 | 0 | 0 | 0 | 0 | 0 | 0 | 1716 |
| 4 | Fujiwara[39] | 2015 | Japan | Single-centered | 0 | 0 | 0 | 857 | 0 | 0 | 0 | 0 | 0 | 10/857 |
| 5 | Ishida[38] | 2015 | Japan | Multi-centered | 2561 | 262 | 504 | 129 | 177 | 838 | 192 | 147 | 573 | 5383 |
| 6 | Koh[37] | 2016 | South Korea | Multi-centered | 18 | 17 | 15 | 4 | 5 | 8 | 3 | 16 | 16 | 102 |
| 7 | Lim[36] | 2017 | Singapore | Multi-centered | 529 | 132 | 110 | 39 | 56 | 65 | 37 | 79 | 77 | 1124 |
| Dental Outcomes | | | | | | | | | | | | | | |
| 1 | Nishimura[35] | 2013 | Japan | Multi-centered | 32 | 7 | 0 | 0 | 0 | 2 | 1 | 1 | 3 | 46 |
| 2 | Kang[34] | 2017 | South Korea | Single-centered | 78 | 22 | 23 | 0 | 0 | 0 | 0 | 18 | 55 | 196 |
| 3 | Tanaka[33] | 2017 | Japan | Single-centered | 42 | 0 | 4 | 1 | 1 | 4 | 2 | 0 | 2 | 56 |
| Hepatic, Gastrointestinal and Renal Outcomes | | | | | | | | | | | | | | |
| 1 | Lee[32] | 2012 | South Korea | Single-centered | 0 | 0 | 0 | 0 | 1 | 10 | 0 | 0 | 0 | 11 |
| 2 | Yoo[31] | 2012 | South Korea | Single-centered | 0 | 0 | 0 | 0 | 0 | 13 | 2 | 0 | 0 | 15 |
| 3 | Watanabe[30] | 2014 | Japan | Single-centered | 7 | 0 | 0 | 0 | 0 | 1 | 0 | 0 | 1 | 9 |
| Immunity Outcomes | | | | | | | | | | | | | | |
| 1 | Mahmoud[29] | 2013 | Japan | Single-centered | 12 | 4 | 4 | 0 | 0 | 10 | 0 | 0 | 0 | 30 |
| 2 | Azanan[28] | 2016 | Malaysia | Single-centered | 81 | 0 | 0 | 0 | 0 | 0 | 0 | 0 | 0 | 81 |
| Mixed Outcomes^ | | | | | | | | | | | | | | |
| 1 | Matsuzaki[27] | 2000 | Japan | Multi-centered | 187 | 0 | 0 | 0 | 0 | 0 | 0 | 0 | 0 | 187 |
| 2 | Han[26] | 2009 | South Korea | Single-centered | 95 | 23 | 35 | 0 | 0 | 13 | 25 | 0 | 50 | 241 |
| 3 | Pakakasama[25] | 2010 | Thailand | Multi-centered | 258 | 0 | 0 | 0 | 0 | 0 | 0 | 0 | 0 | 258 |
| 4 | Han[24] | 2013 | South Korea | Single-centered | 71 | 19 | 30 | 0 | 0 | 10 | 21 | 0 | 42 | 193 |

^ Data extraction on organ-specific toxicities was not conducted on the four studies with mixed outcomes as most lack detailed information on assessment methods for each outcome.

Supplement 2: Quality Assessment of Included Studies

|  |  |  | Objective clearly stated^1^ | Defined study population^2^ | Participation rate >50%^3^ | Inclusion and exclusion criteria^4^ | Sample size justification^5^ | Exposure before outcomes^6^ | Varying amount of exposure^7^ | Exposures clearly defined^8^ | Outcome measures clearly defined^9^ | Outcome assessors blinded^10^ | Key potential confounding variables^11^ | Overall |
| --- | --- | --- | --- | --- | --- | --- | --- | --- | --- | --- | --- | --- | --- | --- |
| Cardiac Outcomes | | | | | | | | | | | | | | |
| 1 | Hamada[80] | 2006 | 1 | 0 | NR | NR | NA | 1 | 1 | 1 | 1 | 1 | 0 ^ | 6 |
| 2 | Cheung[79] | 2011 | 1 | 1 | NR | NR | 0 | 1 | 1 | 1 | 1 | 1 | 0 ^ | 7 |
| 3 | Shimomura[78] | 2011 | 1 | 1 | NR | 1 | 0 | 1 | 1 | 1 | 1 | NR | 0 ^ | 7 |
| 4 | Cheung[77] | 2013 | 1 | 1 | NR | 1 | 0 | 1 | 1 | 1 | 1 | 0 | 1 | 8 |
| 5 | Yu[76] | 2013 | 1 | 1 | NR | 1 | 0 | 1 | 1 | 1 | 1 | 1 | 0 ^ | 8 |
| 6 | Yu[75] | 2013 | 1 | 1 | NR | 1 | 0 | 1 | 1 | 1 | 1 | 1 | 0 ^ | 7 |
| 7 | Cheung[74] | 2015 | 1 | 1 | 1 | 1 | 0 | 1 | 1 | 1 | 1 | NR | 1 | 9 |
| 8 | Li[73] | 2016 | 1 | 1 | NR | 1 | 0 | 1 | 1 | 1 | 1 | NR | 0 ^ | 7 |
| 9 | Li[72] | 2017 | 1 | 1 | NR | 0 | 0 | 1 | 1 | 1 | 1 | NR | 0 ^ | 6 |
| Endocrine, Metabolic, Growth and Fertility Outcomes | | | | | | | | | | | | | | |
| 1 | Yamashita[71] | 2003 | 1 | 1 | NR | 1 | NA | 1 | 1 | 1 | 1 | NR | 0 ^ | 6 |
| 2 | Jaruratanas-irikul[70] | 2004 | 1 | 1 | NR | 1 | NA | 1 | 1 | 1 | 1 | NR | 1 | 8 |
| 3 | Ishiguro[69] | 2007 | 1 | 1 | NR | NR | NA | 1 | 0 | 1 | 1 | NR | 0 ^ | 5 |
| 4 | Miyoshi[68] | 2008 | 1 | 1 | NR | 1 | NA | 1 | 0 | 1 | 1 | NR | 0 ^ | 6 |
| 5 | Adachi[67] | 2009 | 1 | 1 | NR | 1 | NA | 1 | 1 | 1 | 1 | NR | 1 | 8 |
| 6 | Surapolchai[66] | 2010 | 1 | 1 | NR | 1 | NA | 1 | 1 | 1 | 1 | NR | 1 | 8 |
| 7 | Lusawat[65] | 2010 | 1 | 1 | NR | 1 | NA | 1 | 0 | 1 | 1 | NR | 0 ^ | 6 |
| 8 | Tomita[64] | 2011 | 1 | 1 | NR | 1 | NA | 1 | 0 | 1 | 1 | NR | 0 ^ | 6 |
| 9 | Nishi[63] | 2011 | 1 | 1 | NA | NR | NA | 1 | 0 | 1 | 1 | NR | 0 # | 5 |
| 10 | Sohn[62] | 2011 | 1 | 1 | NR | NR | NA | 1 | 0 | 1 | 1 | NR | 1 | 6 |
| 11 | Hyodo[61] | 2012 | 1 | 1 | NR | 1 | NA | 1 | 0 | 1 | 1 | NR | 0 ^ | 6 |
| 12 | Kang[60] | 2012 | 1 | 1 | NR | 1 | NA | 1 | 1 | 1 | 1 | NR | 0 ^ | 7 |
| 13 | Miyoshi[59] | 2012 | 1 | 1 | 1 | 1 | NA | 1 | 0 | 1 | 1 | NR | 0 ^ | 7 |
| 14 | Choi[58] | 2013 | 1 | 1 | NR | 1 | NA | 1 | 1 | 1 | 1 | NR | 1 | 8 |
| 15 | Kojima[57] | 2013 | 1 | 1 | NR | 1 | NA | 1 | 0 | 1 | 1 | NR | 0 ^ | 6 |
| 16 | Han[56] | 2015 | 1 | 1 | 0 | 1 | NA | 1 | 1 | 1 | 1 | NR | 1 | 8 |
| 17 | Lee[55] | 2015 | 1 | 1 | 1 | NR | NA | 1 | 1 | 1 | 1 | NR | 1 | 8 |
| 18 | Adachi[54] | 2017 | 1 | 1 | 1 | 1 | NA | 1 | 1 | 1 | 1 | NR | 0 ^ | 8 |
| 19 | Yoon[53] | 2017 | 1 | 1 | NR | 1 | NA | 1 | 1 | 1 | 1 | NR | 0 ^ | 7 |
| Neurologic and Neurocognitive Outcomes | | | | | | | | | | | | | | |
| 1 | Chan[82] | 2001 | 1 | 1 | NR | 1 | NA | 1 | 1 | 1 | 1 | NR | 1 | 8 |
| 2 | Khong[52] | 2003 | 1 | 1 | NR | 1 | 0 | 1 | 1 | 1 | 1 | NR | 0 # | 7 |
| 3 | Khong[51] | 2004 | 1 | 1 | NR | 1 | NA | 1 | 1 | 1 | 1 | NR | 1 | 8 |
| 4 | Chan[81] | 2006 | 1 | 0 | NR | NR | NA | 1 | 1 | 1 | 1 | NR | 0 | 5 |
| 5 | Khong[50] | 2006 | 1 | 1 | NR | NR | 0 | 1 | 1 | 1 | 1 | NR | 1 | 7 |
| 6 | Akira[49] | 2007 | 1 | 1 | NR | NR | NA | 1 | 0 | 1 | 1 | NR | 0 # | 5 |
| 7 | Chiou[48] | 2009 | 1 | 1 | NR | 1 | NA | 1 | 1 | 1 | 1 | NR | 0 ^ | 7 |
| 8 | Liang[47] | 2013 | 1 | 1 | NR | NR | NA | 1 | 1 | 1 | 1 | NR | 0 ^ | 6 |
| 9 | Kim[46] | 2014 | 1 | 1 | 1 | 1 | NA | 1 | 1 | 1 | 1 | NR | 1 | 9 |
| 10 | Yamasaki[45] | 2015 | 1 | 1 | NR | 1 | NA | 1 | 0 | 1 | 1 | NR | 0 ^ | 6 |
| 11 | Tay[44] | 2016 | 1 | 1 | NR | 1 | NA | 1 | 1 | 1 | 1 | NR | 0 ^ | 7 |
| 12 | Yamasaki[43] | 2017 | 1 | 1 | NR | NR | NA | 1 | 0 | 1 | 1 | NR | 0 ^ | 5 |
| Secondary Malignancy | | | | | | | | | | | | | | |
| 1 | Araki[42] | 2011 | 1 | 1 | NR | 1 | NA | 1 | 0 | 1 | 1 | NR | 1 | 7 |
| 2 | Sun[41] | 2011 | 1 | 1 | 1 | 1 | NA | 1 | 0 | 1 | 1 | NR | 0 ^ | 7 |
| 3 | Ishida[40] | 2013 | 1 | 1 | 1 | 1 | NA | 1 | 1 | 1 | 1 | NR | 1 | 9 |
| 4 | Fujiwara[39] | 2015 | 1 | 1 | NR | NR | NA | 1 | 0 | 1 | 1 | NR | 0 # | 5 |
| 5 | Ishida[38] | 2015 | 1 | 1 | NR | 1 | NA | 1 | 1 | 1* | 1 | NR | 1 | 7 |
| 6 | Koh[37] | 2016 | 1 | 1 | 1 | 1 | NA | 1 | 0 | 1 | 1 | NR | 0 | 7 |
| 7 | Lim[36] | 2017 | 1 | 1 | NR | 1 | NA | 1 | 0 | 1 | 1 | NR | 0 | 6 |
| Dental Outcomes | | | | | | | | | | | | | | |
| 1 | Nishimura[35] | 2013 | 1 | 1 | NR | NR | NA | 1 | 1 | 1 | 1 | NR | 0 ^ | 6 |
| 2 | Kang[34] | 2017 | 1 | 1 | NR | NR | NA | 1 | 1 | 1 | 1 | NR | 1 | 7 |
| 3 | Tanaka[33] | 2017 | 1 | 1 | NR | NR | NA | 1 | 1 | 1 | 1 | NR | 1 | 7 |
| Hepatic, Gastrointestinal and Renal Outcomes | | | | | | | | | | | | | | |
| 1 | Lee[32] | 2012 | 1 | 1 | NR | 1 | NA | 1 | 0 | 1 | 1 | 0 | 0 # | 6 |
| 2 | Yoo[31] | 2012 | 1 | 1 | NR | 1 | NA | 1 | 0 | 1 | 1 | 0 | 0 # | 6 |
| 3 | Watanabe[30] | 2014 | 1 | 0 | 0 | NR | NA | 1 | 0 | 1 | 1 | 0 | 0 # | 4 |
| Immunity Outcomes | | | | | | | | | | | | | | |
| 1 | Mahmoud[29] | 2013 | 1 | 1 | NR | 1 | 0 | 1 | 0 | 1 | 1 | 0 | 0 ^ | 6 |
| 2 | Azanan[28] | 2016 | 1 | 1 | NR | 1 | 0 | 1 | 1 | 1 | 1 | 0 | 1 | 8 |
| Mixed Outcomes^ | | | | | | | | | | | | | | |
| 1 | Matsuzaki[27] | 2000 | 1 | 1 | NR | 1 | NA | 1 | 0 | 1 | 1 | NR | 0 | 6 |
| 2 | Han[26] | 2009 | 1 | 1 | 1 | 1 | NA | 1 | 0 | 1 | 1 | NR | 1 | 8 |
| 3 | Pakakasama[25] | 2010 | 1 | 1 | NR | NR | NA | 1 | 0 | 1 | 1 | NR | 0 | 5 |
| 4 | Han[24] | 2013 | 1 | 1 | 1 | 1 | NA | 1 | 0 | 1 | 1 | NR | 1 | 8 |

NA: Not applicable; NR: Not reported

*Some do not have treatment data and were excluded from multivariable analysis

^ Only univariate analysis conducted

# sample size is probably too small for multivariable analysis

There is no standard grading criteria to define “high”, “moderate”, or “weak” quality. We have defined “high” quality as a score of 8 or more, “moderate” quality as a score of 6 to 7, and “low” quality as a score of 5 or less.

Description of assessment criteria:

1. Research question: All included studies have explicitly defined a research question.
2. **Study population: Studies will be rated “1” if authors specified** demographics, location, and time period of the study sample.
3. **Participation rate of at least 50%: Studies will be rated “1” if authors reported the participation rate. However, the majority of the studies either did not report the participation rate, or selected their sample from an in-house database or cancer registry.**
4. Inclusion and exclusion criteria developed prior to recruitment or selection of the study population: Studies will be rated “1” if authors specified the criteria, or reasons for excluding certain subjects. Studies will be rated “0” if authors only stated the source of the sample without stating the underlying criteria for selecting the sample.
5. **Sample size justification: All the included studies did not include sample size justification. However, the majority of the studies were rated as “NA” because they are descriptive in nature. Studies will be rated “0” if hypothesis testing was conducted between groups (eg: cancer survivors versus controls) but sample size justification was not provided.**
6. **Exposure (cancer and cancer treatment) assessed prior to outcome measurement (cost-treatment clinical outcome): Due to the nature of our research question, all the included studies were rated as “1”. Our selection criteria of the studies for this review have excluded those studies where clinical outcomes were assessed during active treatment.**
7. **Different levels of the exposure (treatment intensity) of interest: Studies were rated as “1” if treatment is defined and analyzed in continuous variable (eg. radiation dose, cumulative chemotherapy dose), or intensity level (eg: brain radiation of >18Gy vs < 18 Gy).**
8. **Exposure measures and assessment: Due to the nature of our research question, all the included studies have clearly defined exposure, which refer to cancer and cancer treatment.**
9. **Outcome measures: All the included studies were rated “1” because the selection criteria of the studies for this review only included those with objective clinical measures. Therefore, the outcomes measures were clearly defined in all studies.**
10. **Blinding of outcome assessors: Most of the studies either did not report the blinding of the assessors, not blinding is not relevant because the entire cohort received the exposure. However, some studies that involved imaging were rated as “1” if authors have specified the blinding of the radiologists in the study.**
11. Key potential confounding variables measured and adjusted for, such as by statistical adjustment for baseline differences: Studies were rated as “1” if multivariable analysis was conducted, taking into account clinically relevant covariates. Studies were rated as “0” if only univariate analysis was conducted, or the sample size is probably too small for multivariable analysis

There were 3 criteria that were part of the Quality Assessment Tool for Observational Cohort and Cross-Sectional Studies, but were excluded from this quality assessment exercise:

1. **Sufficient timeframe to see an effect: Different clinical outcomes may have different onset. For example, cisplatin-related hearing impairment may occur immediately post-treatment and persist till long-term survivorship, while cardiovascular morbidities typically occur more than 2 years post-treatment. Hence, it is difficult to determine the sufficient timeframe to see the clinical outcome.**
2. **Repeated exposure assessment: This is not relevant as the exposure, which refers to the cancer and cancer treatment occur at a single single time point and are captured only once.**
3. **Follow-up rate: Most studies did not report follow-up rate from baseline, which in this case may refer to the survival rate, or survivors who were lost to follow up.**

Reference: National Heart, Lung and Blood Institute. Quality Assessment Tool for Observational Cohort and Cross-Sectional Studies. URL: <https://www.nhlbi.nih.gov/health-topics/study-quality-assessment-tools>. Accessed on 1 July 2018.

Supplement 3: Studies with Other Outcomes (Dental, Hepatic, Gastrointestinal and Renal, and Immunity Outcomes)

| Author | Country | N | Sex (% male) | Diagnoses* | Age (Dx) | Age (follow-up) | Follow-up time | Treatment Modality | | | Outcome assessments | Prevalence/ Result | Risk factors |
| --- | --- | --- | --- | --- | --- | --- | --- | --- | --- | --- | --- | --- | --- |
| Dental | | | | | | | | | | | | | |
|  |  |  |  |  |  |  |  | Chemo | Rad | HCST |  |  |  |
| Nishimura[35] | Japan | 46 | 59 % | Mixed | 3.7  [0-9.8] | 17.7  [7.0-32.2] | NR | ✓  (100%) | ✓  (50%) | ✓  (44%) | Tooth formation anomalies: abnormal root development, microdonts, tooth agenesis, tooth formation anomaly | - Tooth formation anomalies (89.1%) | - Young age at diagnosis (≤8 years) - Use of alkylators, especially busulfan |
| Kang[34] | Korea | 196 | 65 % | Mixed | 4.7  [0 - 16.4] | 14.9  [4.6-33.9] | 6.9  [2.1.-22.5]  post-tx | ✓  (99%) | ✓  (32%) | ✓  (24%) | Tooth agenesis, microdontia, V-shaped roots, taurodontism, abnormal root development | - Exhibited at least one dental anomaly (55.6%) | - Young age at diagnosis (≤3 years) - Hematopoietic stem cell transplantation - Multiple classes of chemotherapeutic agents (≥4 classes) - Heavy metal agents |
| Tanaka[33] | Japan | 56 | 45 % | Mixed | 1.9  [0-13.7] | 13.9  [4.6-32.7] | ≥3 years post-tx or 5 years post-dx | ✓  (98%) | ✓  (39%) | ✓  (46%) | Oral and maxillofacial abnormalities: hypodontia, abnormal roots, enamel defects, microdontia | - Oral or maxillofacial abnormalities (73.2%) | - Treated at age older than 4 years - Hematopoietic stem cell transplantation |
| Hepatic, Gastrointestinal and Renal | | | | | | | | | | | | | |
|  |  |  |  |  |  |  |  | Chemo | Rad | HCST |  |  |  |
| Lee[32] | Korea | 11 | 36% | Mixed | 1.9  [0.8-6.5] | 9  [4.0-12.3] | 5.8  [3.2-9.5]  post-dx | ✓  (100%) | ✓  (91%) | ✓  (91%) | Liver CT | - 22 nodules in all (100%) patients | - VOD - Cytoreductive agents |
| Yoo[31] | Korea | 15 | 33% | Mixed | 2.7  [0.5-10] | 12.7  [8-20] | 8.5  [4.5-13.5]  post-dx | ✓  (NR) | ✓  (87%) | ✓  (80%) | Liver CT, ultrasound | - 43 nodules in all (100%) patients | - Female |
| Watanabe[30] | Japan | 9 | 78% | Mixed | 6  [1-17] | 6  [1-17] | 11.7  [6.6-14.2]  post-dx | ✓  (100%) | ✓  (100%) | ✓  (100%) | eGFR | - Decreased but normal eGFR (44.4%) - Hypertension (11%) | - TBI schedule of 12 Gy in 6 fractions over three consecutive days |
| Immunity | | | | | | | | | | | | | |
|  |  |  |  |  |  |  |  | Chemo | Rad | HCST |  |  |  |
| Mahmoud[29] | Japan | 30 | 60% | Mixed | [1.0-16.4]  age at last chemotherapy | 8.0  [6.0-18.0] | 5.3  [3.8-10.5]  post-tx | ✓  (100%) | NR | NR | Quantification of EBV DNA | - Continuous detection of EBV DNA (20%) | - >5 years after treatment - ALL and lymphoma - Age 5 to 10 years old at follow up |
| Azanan[28] | Malaysia | 81 | 42% | Leukemia | 5  [3-9] | 26  [22-30] | 19  [14-22]  post-tx | ✓ (100%) | ✓ (52%) | NR | CMV serology, CMV-specific immunity, cytokine, inflammatory markers | - Elevated levels of interleukein-6, human C-reactive protein and CMV immune activation, as compared to controls. | - Prior anthracyclines and radiotherapy exposure |

*Breakdown of cancer diagnoses are presented in Table 1

Studies are arranged in chronological order.

CMV, cytomegalovirus; CT, computed tomography; Dx, diagnosis; EBV, Epstein–Barr virus; GFR, estimated glomerular filtration rate; NR, not reported; TBI, total body irradiation; tx, treatment; VOD, veno-occlusive disease

Figure 1: Barriers and Evidence-based Enablers of a Comprehensive Survivorship Program


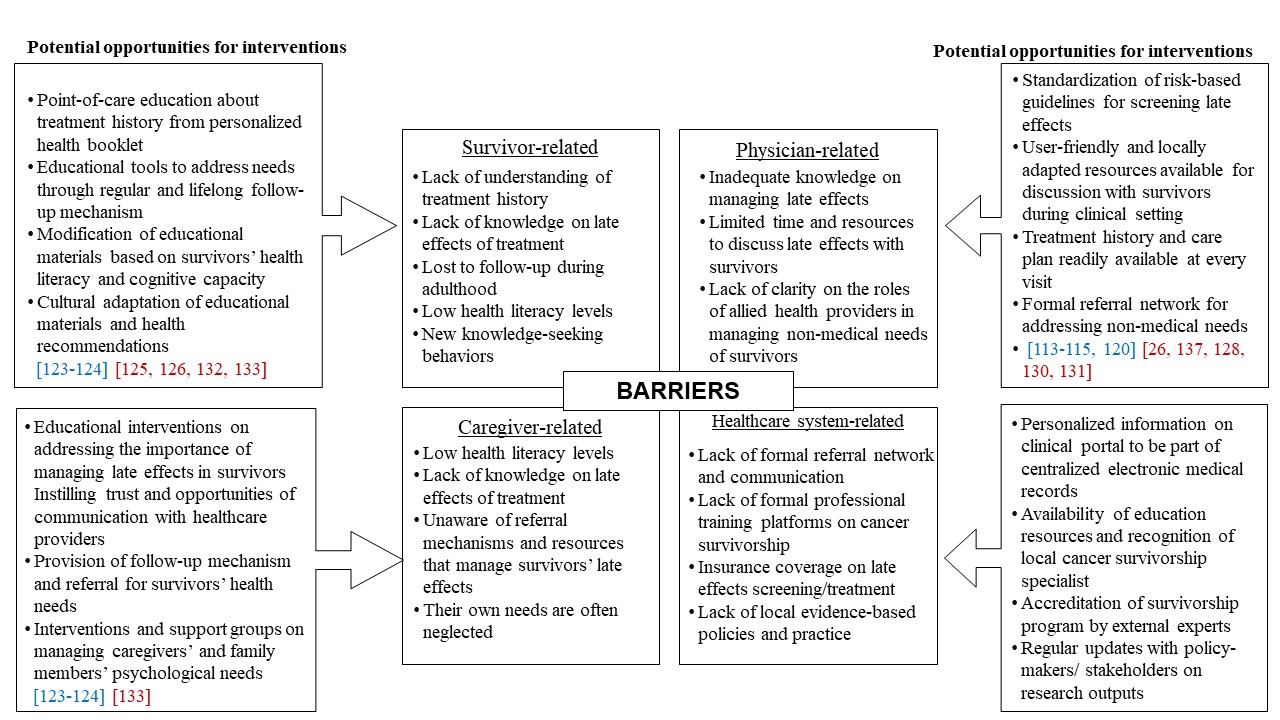


Legend:

Citations in blue are evidence gathered from the non-Asian population (eg: Western population).

Citations in red are evidence gathered from the Asian population
